# Hospitals and the Spirit Tax

**Published:** 1915-07-24

**Authors:** N. Bishop Harman

**Affiliations:** 108 Harley Street, W.


### THE EDITOR'S LETTER-BOX.

### Hospitals and the Spirit Tax.

*To the Editor of* The Hospital.

Sir,—I could have wished that you had been informed of the latest phase of this question before your leading article of the 17th inst. was written, for then I feel sure you would have approved as warmly of the action of the British Medical Association as in your incomplete knowledge you condemn. As Chairman of the Parliamentary Sub-Committee of the Association which dealt with this matter, I trust that I may be permitted to reply to your criticism.

When the second reading of the Finance (No. 2) Bill was taken, there appeared a new official clause extending the provisions of 2 Edw. VII., c. 7 (authorising the use of duty-free alcohol), to hospitals, and these institutions were defined in a special section. The appearance of this clause was sudden, no prior warning having been given that it would appear in that Bill. The representative organisations of the two professions naturally concerned in a matter of this kind accordingly asked for delay on certain grounds which were represented to the Chancellor of the Exchequer. The clause was withdrawn, and forthwith Mr. Bridgeman, the Junior Lord of the Treasury, asked representatives of the two Associations to meet him, with the intention of devising some form of words that would relieve the difficulties foreshadowed. Mr. Bridgeman, of course, understood that the opposition of the two Associations was not to the principle of giving a concession to hospitals, but to the method by which this concession was to be given. At the interview we put before him our criticism of the official clause and suggested alternative procedures. The fact that Mr. Bridgeman accepted our suggestions, so far as he himself was concerned, seems to justify our action. He has since written stating that the suggestions will be adopted officially, but that, seeing the new clauses will necessitate recommittal of the Bill, their presentation must await for the next Finance Bill.

Our criticism briefly was on two grounds: first, that it was bad policy to base a grant to a hospital on the amount of alcohol which it could contrive to consume—that would have been the effect of the clause that was withdrawn. Secondly, we pointed out that the whole question of duty-free alcohol was one which had exercised the minds of the medical profession for a long time, and could not rightly be dealt with in this summary fashion. In addition, we pointed out that the official definition of a hospital opened the door to widespread abuse in the formation of one-man "hospitals," and that a later addition to the official clause limiting the benefit to institutions not run for gain was insufficient to shut out the possibility of abuse.

Our constructive suggestions were three:—

1. That a full and proper inquiry should be held into the best way of dealing with the subject of duty-free alcohol in medicine.

2. That meantime a fixed grant should be made to duly recognised hospitals equal to the tax on all the alcohol used by them for medical purposes in the year 1914.

3. That an Advisory Committee to the Commissioners of Customs and Excise should be appointed to which all applications from hospitals claiming the benefit of the Act should be sent for certification of eligibility.

It should hardly be necessary in a medical paper to point out the *prima facie* absurdity of the suggestion that the British Medical Association, of all bodies, should be inimical to any judicious concession to hospitals; but if the point needs emphasis, may I add that the whole profession, hospitals included, have to thank the Association in cooperation with the Pharmaceutical Society, that medicine is exempt from the new eighteen-penny tax on raw alcohol? I do not doubt that the action of the Association on this further and larger matter will be as fruitful of good.—I am, yours faithfully,

N. Bishop Harman, F.R.C.S.

108 Harley Street, W.,
July 20, 1915.

[We thank Mr. Harman for his letter. We may point out, however, that the present methods of the B.M.A. seem invariably to lead to prolonged delays. If reasonable improvement resulted from such delays they might be tolerated. But do they in the case of the B.M.A.? The difficulties of definition are not insurmountable as Mr. N. B. Harman seems to think.—Ed. The Hospital.]

